# Concurrent and predictive validity of the infant motor profile in infants at risk of neurodevelopmental disorders

**DOI:** 10.1186/s12887-021-02522-5

**Published:** 2021-02-06

**Authors:** Riccardo Rizzi, Valentina Menici, Maria Luce Cioni, Alessandra Cecchi, Veronica Barzacchi, Elena Beani, Matteo Giampietri, Giovanni Cioni, Giuseppina Sgandurra, the Clinical CareToy-R Consortium, Claudia Artese, Marta Cervo, Carlo Dani, Patrizio Fiorini, Viola Fortini, Simona Giustini, Clara Lunardi, Giada Martini, Martina Orlando, Letizia Padrini, Filomena Paternoster

**Affiliations:** 1 Tuscan PhD Programme of Neuroscience, University of Florence, Pisa and Siena, Florence, Italy; 2 Department of Developmental Neuroscience, IRCCS Fondazione Stella Maris, Pisa, Italy; 3 Neonatal Intensive Care Unit, Children’s Hospital A. Meyer, Florence, Italy; 4 Division of Neonatology, Careggi University Hospital, University of Florence, Florence, Italy; 5 Neonatal Intensive Care Unit, Pisa University Hospital Santa Chiara, Pisa, Italy; 6 Department of Clinical and Experimental Medicine, University of Pisa, Pisa, Italy

**Keywords:** Infant motor profile, Alberta infant motor scale, Neurodevelopmental disorder, general movement assessment

## Abstract

**Background:**

Preterm infants and infants with perinatal brain injury show a higher incidence of neurodevelopmental disorders (NDD). The Infant Motor Profile (IMP) is a clinical assessment which evaluates the complexity of early motor behaviour. More data are needed to confirm its predictive ability and concurrent validity with other common and valid assessments such as the Alberta Infant Motor Scale (AIMS) and Prechtl’s General Movement Assessment (GMA). The present study aims to evaluate the concurrent validity of the IMP with the AIMS, to assess its association with the GMA, to evaluate how the IMP reflects the severity of the brain injury and to compare the ability of the IMP and the AIMS to predict an abnormal outcome in 5-month-old infants at risk of NDD.

**Methods:**

86 infants at risk of NDD were retrospectively recruited among the participants of two clinical trials. Preterm infants with or without perinatal brain injury and term infants with brain injury were assessed at 3 months corrected age (CA) using the GMA and at 5 months CA using the IMP and the AIMS. The neurodevelopmental outcome was established at 18 months.

**Results:**

Results confirm a solid concurrent validity between the IMP Total Score and the AIMS (Spearman’s ρ 0.76; *p* < .001) and a significant association between IMP Total Score and the GMA. Unlike the AIMS, the IMP Total score accurately reflects the severity of neonatal brain injury (p < .001) and proves to be the strongest predictor of NDD (*p* < .001). The comparison of ﻿areas under receiver operating characteristic curves (AUC) confirms that the IMP Total score has the highest diagnostic accuracy at 5 months (AUC 0.92). For an optimal IMP Total Score cut-off value of 70, the assessment shows high sensitivity (93%) and specificity (81%) (PPV 84%; NPV 90%).

**Conclusions:**

Early motor behaviour assessed with the IMP is strongly associated with middle-term neurodevelopmental outcome. The present study confirms the concurrent validity of the IMP with the AIMS, its association with the GMA and its ability to reflect brain lesion load, hence contributing to the construct validity of the assessment.

**Trial registration:**

NCT01990183 and NCT03234959 (clinicaltrials.gov).

**Supplementary Information:**

The online version contains supplementary material available at 10.1186/s12887-021-02522-5.

## Background

Over the last decades, the increasing survival rates of preterm and high-risk full-term infants is becoming a reason for growing concern regarding their neurodevelopmental outcome. Consequences may include different forms of neurodevelopmental disorders (NDD). The term NDD includes a wide range of neurological and psychiatric conditions such as cerebral palsy (CP), social communication disorder, attention deficit hyperactivity disorder (ADHD), and brain malformations, resulting from a precocious disruption of functional brain connectivity [1]. Early detection of NDD is becoming one of the greatest challenges in developmental neurology since early evidence seems to indicate that response to an intervention is more effective if provided during early infancy, when brain plasticity is at its highest levels [2].

It is widely accepted that standardized follow-up programs are crucial for the early detection of NDD; nevertheless, the identification of the right diagnostic instruments to be used at the right time is still a matter of debate. Indeed, an ideal clinical instrument should be able to detect early signs of atypical development and to predict the severity of the outcome. To date, a substantial number of neuromotor assessments have been proposed. Among them, Prechtl’s General Movements Assessment (GMA) proved to be highly reliable in the prediction of long term neurologic dysfunctions such as CP during the first months of life [3, 4]. Accumulating evidence suggests that the GMA has the strongest accuracy in the prediction of later cognitive dysfunction, further supporting the use of this tool in the early assessment of infants at risk of NDD [5, 6]. The GMA is based on a standardized qualitative analysis of infant’s spontaneous motor repertoire in which factors such as variability, distribution and complexity of movements reflect the pattern of typical and atypical development. However, after 4 to 5 months post-term age spontaneous general movements gradually fade-out, leaving room for a new complex repertoire of intentional goal-directed movements. At that age the GMA cannot be performed and, for this reason, there is a need for other standardized assessment tools which will provide insight, not only into the presence of specific neurological signs but also into the quality and variability of motor behaviour.

A growing amount of literature seems to indicate that instruments assessing the *quality* of motor behaviour can provide more subtle information about the brain functioning of infants rather than a traditional neurological evaluation [7]. In general, the evaluation of quality and especially variability of the early motor repertoire seems to reflect brain functional integrity and connectivity in a much more accurate way. As a result, these kinds of qualitative assessments turned out to be useful, not only for the prediction of major motor disorders (such as CP), but also for the identification of early signs of other NDD [4, 8].

In this framework, the Infant Motor Profile (IMP) assessment has been developed [9]. The IMP is a video-based assessment of motor behaviour of infants from 3 months of corrected age (CA) until the age of autonomous walking (approximately 18 months).

The IMP was created in line with the Neuronal Group Selection Theory (NGST). According to this theory, infant motor development is characterized by two phases of variability: a first phase of abundant variation of movements and exploration of all motor possibilities, and a second phase during which infants learn to select the most adaptive strategies out of a motor repertoire based on trial-and-error experiences [10]. As a consequence, an early brain lesion results in a limitation of both phases leading to a reduction in the variation of the motor repertoire and to problems with the selection of the most adaptive motor behaviour [11].

Consistent with this framework, the IMP has been developed on the assumption that qualitative aspects of movement are much more informative than the mere achievement of motor milestones [12]. A description of the IMP is provided in the Methods section. After the first report by Heineman et al. (2008), the authors reported a strong correlation between the IMP and other widely used assessment tools such as the Alberta Infant Motor Scale (AIMS) and a satisfactory inter-rater reliability [13]. Subsequently, they explored the association between the IMP values and later cognitive and motor impairment. In 2011, they longitudinally assessed a group of preterm and full-term infants using the IMP at 4, 6, 10 and 12 months showing a high ability to predict CP at 18 months [14]. Recently, the same group demonstrated a clear relationship between developmental motor trajectories measured with the IMP and later outcome at school age [15]. These findings support the idea that the variability of an early motor repertoire could represent not only an early marker of major motor disorders but also of neurodevelopmental disorders as a whole. Nevertheless, these studies mainly involved infants being at relatively low risk for NDD (e.g. children of parents with reduced fertility or term infants with no additional risk factors) [12, 14–16] raising the need to explore the relation between the IMP and outcome in high risk populations. Moreover, as neonatal brain ultrasound and MRI is becoming increasingly important in the prognosis of at-risk infants, the relation between the imaging findings and the IMP still needs to be fully elucidated. Finally, optimal cut-off scores have, as yet, not been established.

The aims of the present study were firstly to confirm the concurrent validity of the IMP with the AIMS in a selected population of infants at risk of NDD, secondly, to evaluate its association with the GMA, thirdly, to investigate how the IMP reflects the severity of the brain injury and finally to compare the predictive ability of the IMP and the AIMS in a population of selected infants with an increased risk of NDD.

## Methods

### Participants

For the present retrospective study, we screened for possible inclusion, 99 participants of two clinical trials which included a population at risk for NDD (ClinicalTrials identifier NCT01990183, NCT03234959). Both trials were approved by the Tuscan Region Paediatric Ethics Committee. The first RCT (NCT01990183) investigated the effect of a 4-week-long intervention program with CareToy in preterm infants. The inclusion criteria were a gestational age between 28 + 0 weeks and 32 + 6 weeks, and an age at first assessment between 3 and 9 months. The exclusion criteria defined were: the presence of brain injury, infants born small for gestational age, history of seizures, severe sensory loss, and other polymalformative syndromes. The second RCT (NCT03234959) compared the effect of an 8-week-long intervention program with a revised version of CareToy (CareToy-R) and Infant Massage in infants with perinatal brain injury [17]. Infants with the following criteria were included: the presence of abnormal neurological signs at 2–4 months CA, the presence of early brain injury, severe sensory loss, progressive neurological disorders, malformation of CNS, polymalformative syndromes.

For the purpose of the present retrospective study, only those infants who fulfilled the following criteria were selected: age at GMA 3 months, age at the IMP and the AIMS assessments 5 months, follow-up visit at 18 months. Following the exclusion of 13 infants (9 infants from the first RCT and 4 from the second RCT) as they did not meet the inclusion criteria, a total number of 86 infants (52 from the first RCT and 34 from the second RCT) were included in the present study. A flowchart showing the process of how the enrolment of the participants in the study was conducted is provided as Supplementary Material.

### Data collection and measurements

All the subjects were recruited during hospitalization in the NICUs or during the follow-up programs for high risk infants at three different referral centres: the Neonatal Intensive Care Unit of the “University Hospital Santa Chiara” in Pisa, the Neonatal Intensive Care Unit of “Meyer Children’s Hospital” in Florence and the Neonatal Intensive Care Unit of “Careggi University Hospital” in Florence. Written informed consent forms were signed by parents or the legal representative of the eligible infants.

After discharge from the NICU, all the patients were assessed at 3 months, 5 months and 18 months of CA. At 3 months of CA, Prechtl’s Assessment of General Movements (GMA) of pre-recorded videos was performed independently by two experienced assessors certified by the GMs Trust (GC and AG). Physiologic fidgety movements were classified as normal (normal fidgety movements) or not normal (absent fidgety, sporadic fidgety, abnormal fidgety movements) [18]. Whenever disagreement arose between the two assessors, the video was discussed until agreement on a final score was reached.

At 5 months CA, all infants were assessed with the IMP and the Alberta Infant Motor Scale (AIMS). The IMP allows to assess infant motor behaviour in different conditions. The assessment consists of a video-recording of approximately 15 min which is intended to evaluate spontaneous motor behaviour in different positions (supine, prone, sitting, standing and walking). Subsequently, reaching, grasping and manipulations are assessed with the presentation of interesting objects in a supine and supported sitting position. No strict order of administration is required so that the assessment can adapt to the infant’s age, preferences and interests [9]. 80 items are then scored off-line, based on the video-recording on a dedicated scoresheet. The items are classified into four qualitative domains (Variation, Adaptability, Fluency and Symmetry) and one quantitative domain (Performance). The first and second domains reflect the two phases of NGST: notably, the Variation domain refers to the size of the motor repertoire while the Adaptability domain refers to the ability of performing a selection of motor strategies from the entire repertoire. The Fluency domain contains items that assess the ability of the infant to adjust and calibrate movements and to fine-tune movements, the Symmetry domain investigates the presence of stereotyped asymmetric movements and the Performance domain is focused on achievements of motor milestones.

The AIMS is a standardised scale designed to evaluate gross-motor abilities in infants [19]. The assessment, which has a high sensitivity, specificity and accuracy in detecting motor deficits in infants [20, 21], consists of 58 items which assess motor skills in prone, supine, sitting and standing positions. Each item can be scored as ‘observed’ or ‘not observed’; the sum of the observed items provides a global score which is plotted on a percentile motor growth curve in order to determine motor performance percentiles compared to the normative sample of infants of the same age.

All the clinical assessments were video recorded and subsequently scored off-line by a trained assessor (VM) who was blind to the treatment. As previously described by Heineman and et al., since infants under the age of 6 months show limited ability to select appropriate strategies from the motor repertoire, the Adaptability domain was not assessed [9, 11].

The final outcome was determined at 18 months CA after a clinical neurodevelopmental assessment was performed by a child neurologist (RR) who was blind to the assigned treatment. Additional clinical assessments (Bayley-III, ADOS-2 …) were individually chosen according to the clinical picture. The presence of a NDD was defined according to the DSM5 criteria by the presence of a significative impairment in motor, cognitive or social functions including CP, global developmental delay, social communication disorders, behavioural disorders, fine motor and coordination dysfunctions [22].

Serial cranial ultrasound scans (cUS) were performed in the NICUs. When the cUS was suggestive of brain injury, the infants were further investigated with brain magnetic resonance imaging (MRI). Term and preterm infants who showed any sign of neurological diseases (hypoxic-ischemic encephalopathy, stroke, seizures …) were scanned with MRI as part of the standard clinical care. cUS and MRI images were evaluated in order to provide an overall stratification between: a) absence of lesions; b) mild/moderate brain injury (preterm white matter injury grade I-II [23], intraventricular haemorrhages grade I-III [24], hypoxic-ischemic injury with predominant watershed pattern [25], ischemic stroke without basal ganglia involvement, small unilateral haemorrhagic infarction); c) severe injury (preterm white matter injury grade III, hypoxic-ischemic injury with predominant basal ganglia-thalami pattern, extensive bilateral haemorrhagic infarction, ischemic stroke with basal ganglia involvement or asymmetry of the posterior limb of the internal capsule).

### Statistical analysis

A statistical analysis was performed using ﻿IBM SPSS Statistics 25.0 for Mac (IBM Corporation, Armonk, NY). Demographic and clinical summaries (sex, gestational age, brain injury and GMA) were computed for each subgroup. The normality of data distribution was verified by Shapiro-Wilk’s Test while non-parametric analyses were used to verify the non-normal distribution of the majority of the data. When conducting the concurrent validity analysis, Spearman’s rank correlation coefficient (ρ) was calculated to examine the association between the IMP scores and the AIMS scores. Correlation was defined as strong for values of ρ > 0.75, moderate for values of ρ 0.50–0.75, fair for values of ρ 0.25- < 0.50, weak for values of ρ < 0.25 [26]. The distribution of the IMP and AIMS values in relation to the GMA was evaluated with the Mann-Witney test. The association between the IMP scores and the severity of the brain injury was assessed using the Kruskal-Wallis test followed by a pairwise multiple comparison of mean ranks. Significance values were adjusted by the Bonferroni correction for multiple tests. Correlations between the IMP scores, the AIMS scores and the clinical outcome were tested for the prediction analysis with the Mann-Witney test; individual U coefficients were reported separately for each domain. A binary logistic regression model was used to estimate the ability of the IMP total score and the AIMS score to predict the outcome by applying the forced entry method. The Hosmer-Lemeshow test was used to determine the goodness of fit. The predictive power of the model was calculated from the Nagelkerke’s R2 and the overall accuracy of the classification.

Finally, receiver operating characteristic (ROC) curves were computed to assess the individual predictive ability of both the IMP and the AIMS and to provide possible optimal cut-off points at 5 months CA for the prediction of NDD. Values of areas under the ROC curve (AUC) of 0.50 suggested no diagnostic accuracy of the test, values of 0.50–0.70 were considered to indicate poor discrimination, values of 0.70–0.80 were considered acceptable, 0.80–0.90 was regarded as excellent; values over 0.90 were considered outstanding [27]. Differences and correlations with *p* < .05 were considered statistically significant.

## Results

The mean gestational age of the study sample was 32 weeks (range 24 + 5–40 + 5; SD 3.9). The mean age at the IMP assessment was 4.9 months (range 4.0–6.0; SD 0.63). 34 infants presented perinatal brain injury (namely haemorrhagic infarctions, stroke or preterm white matter injury). The clinical characteristics of the study sample are presented in Table 1. At 3 months 33 infants (38.4%) showed sporadic or absent fidgety movements at the GMA; no abnormal fidgety movements were reported. A high interscorer agreement was reached among the assessors on the first evaluation of GMs (Cohen’s kappa = 0.80), while agreement was reached for the totality of the assessments following discussion. All the infants included in the study completed the follow-up at 18 months CA. At the end of the study 27 patients (31.4%) presented a NDD, and 59 patients (68.6%) were considered to be typical. Among the 27 infants with NDD, the prevalent diagnosis was CP in 14, followed by minor motor disorders in 6, cognitive impairments in 5, social communication disorders in 2.

**Table 1:** Demographic and clinical characteristics of the study sample

	Typical, n (%) ***n*** = 59	NDD, n (%) ***n*** = 27
**Sex**		
Male (*n* = 46)	34 (73.9%)	12 (26.1%)
Female (*n* = 40)	25 (62.5%)	15 (37.5%)
**Gestational Age**		
25–31 weeks (*n* = 44)	33 (75.0%)	11 (25.0%)
32–36 weeks (n = 27)	21 (77.8%)	6 (22.2%)
37–41 weeks (*n* = 15)	5 (10.0%)	10 (90%)
**Brain injury**		
No brain injury (*n* = 52)	49 (94.2%)	3 (5.8%)
Mild/moderate injury (*n* = 14)	8 (57.1%)	6 (42.9%)
Severe brain injury (*n* = 20)	2 (10.0%)	18 (90.0%)
**GMA**		
Normal Fidgety (*n* = 53)	49 (92.5%)	4 (7.5%)
Not Normal (Absent / Sporadic) Fidgety (*n* = 33)	10 (30.3%)	23 (69.7%)

### Concurrent validity of the IMP with the AIMS

A clear and statistically significant relation between the IMP values and the AIMS total values was evident for the IMP total score and for almost all of the domain scores. The IMP Total and Performance domains showed a strong correlation with the AIMS (Spearman’s *ρ* 0.76 and 0.89 respectively; *p < .*001) while there was a moderate correlation between the IMP Variation and Symmetry and the AIMS (Spearman’s *ρ* .58 and .56 respectively; *p* < .001).

### IMP assessment and GMA

**Fig. 1:**
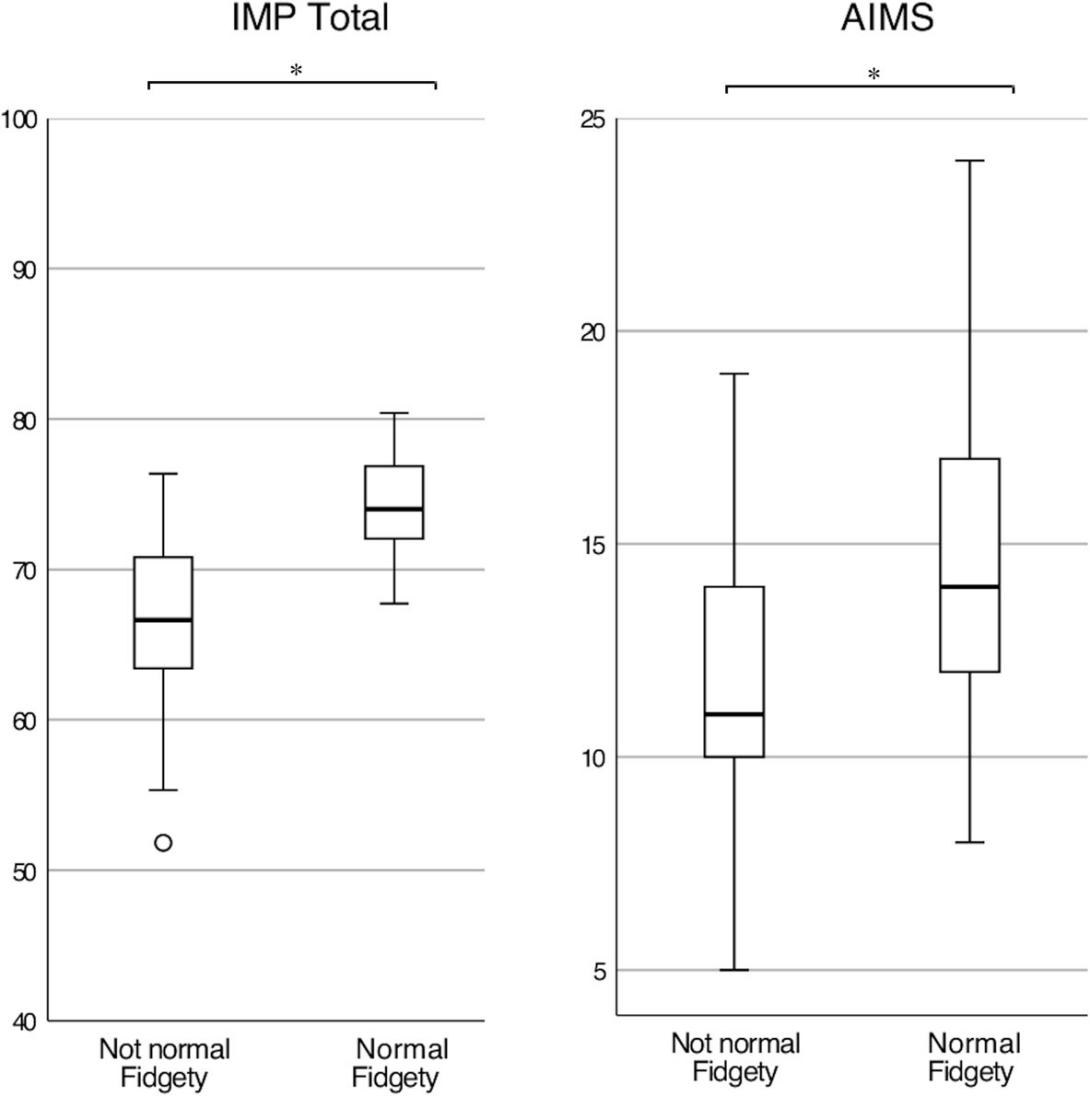
Association between Prechtl’s General Movement Assessment at 3 months and the Infant Motor Profile Total and the Alberta Infant Motor Scale at 5 months corrected age. * *p* < .001

The distribution of the IMP Total scores proved to be significantly different among infants with normal and not normal fidgety movements at the GMA (Mann-Whitney U = 83; *p* < .001) suggesting a strong association between the two assessments (Fig. 1). The distribution of the AIMS values showed a weaker association (Mann-Whitney U = 235; *p* < .001).

### Correlation between the IMP and the AIMS with neuroimaging data

Both the IMP Total (*p* < .001) and the AIMS (*p* < .05) scores correlated with the presence and severity of the brain injury at the neonatal brain MRI (Table 2). All of the IMP domain scores showed an individual correlation with the severity of the lesion load (Variation, Symmetry, Performance *p* = <.001; Fluency *p* < .05). The post-hoc analysis for each group showed a significant correlation for the IMP Total score only (*p* < .001).

**Table 2:** Distribution of scores among MRI severity classes

	No brain lesions median (interquartile range)	Mild/Moderate injury median (interquartile range)	Severe injury median (interquartile range)	***p*** value	
**IMP Total Score**	74.2 (4.7)	71.4 (4.2)	64.4 (7.0)	<.001	*
IMP Variation	71.0 (7.9)	70.1 (7.0)	62.5 (6.3)	<.001	
IMP Fluency	75.0 (−)	75.0 (−)	75.0 (−)	.007	
IMP Symmetry	100 (4.8)	91.9 (5.9)	77.0 (22.6)	<.001	
IMP Performance	55.1 (9.3)	49.9 (10.6)	45.9 (10.8)	<.001	
**AIMS Total Score**	14.0 (5)	13.0 (5)	10.0 (4)	<.05	

* Post-hoc pairwise comparison analysis significant at *p* < .05. *IQR* interquartile range

### Predictive validity of the IMP and the AIMS

Distribution of IMP and AIMS scores compared to the outcome at 18 months are reported in Table 3.

**Table 3:** Distribution of scores at 5 months in infants with typical development and neurodevelopmental disorders (NDD). *p* values and *U* coefficients of the Mann-Whitney *U* test

	Typical Median (interquartile range)	NDD Median (interquartile range)	p value	U coefficient
**IMP Total Score**	74.0 (4.6)	65.6 (9.1)	< 0.001	83
**IMP Variation**	71.4 (8.3)	62.5 (6.2)	< 0.001	254
**IMP Fluency**	75.0 (−)	75 (−)	0.01	646
**IMP Symmetry**	100 (4.8)	83.3 (23.8)	< 0.001	131
**IMP Performance**	54.8 (9.3)	45.9 (8.1)	< 0.001	309
**AIMS Total Score**	14.0 (5.0)	11.0 (2.0)	< 0.001	234

**Fig. 2:**
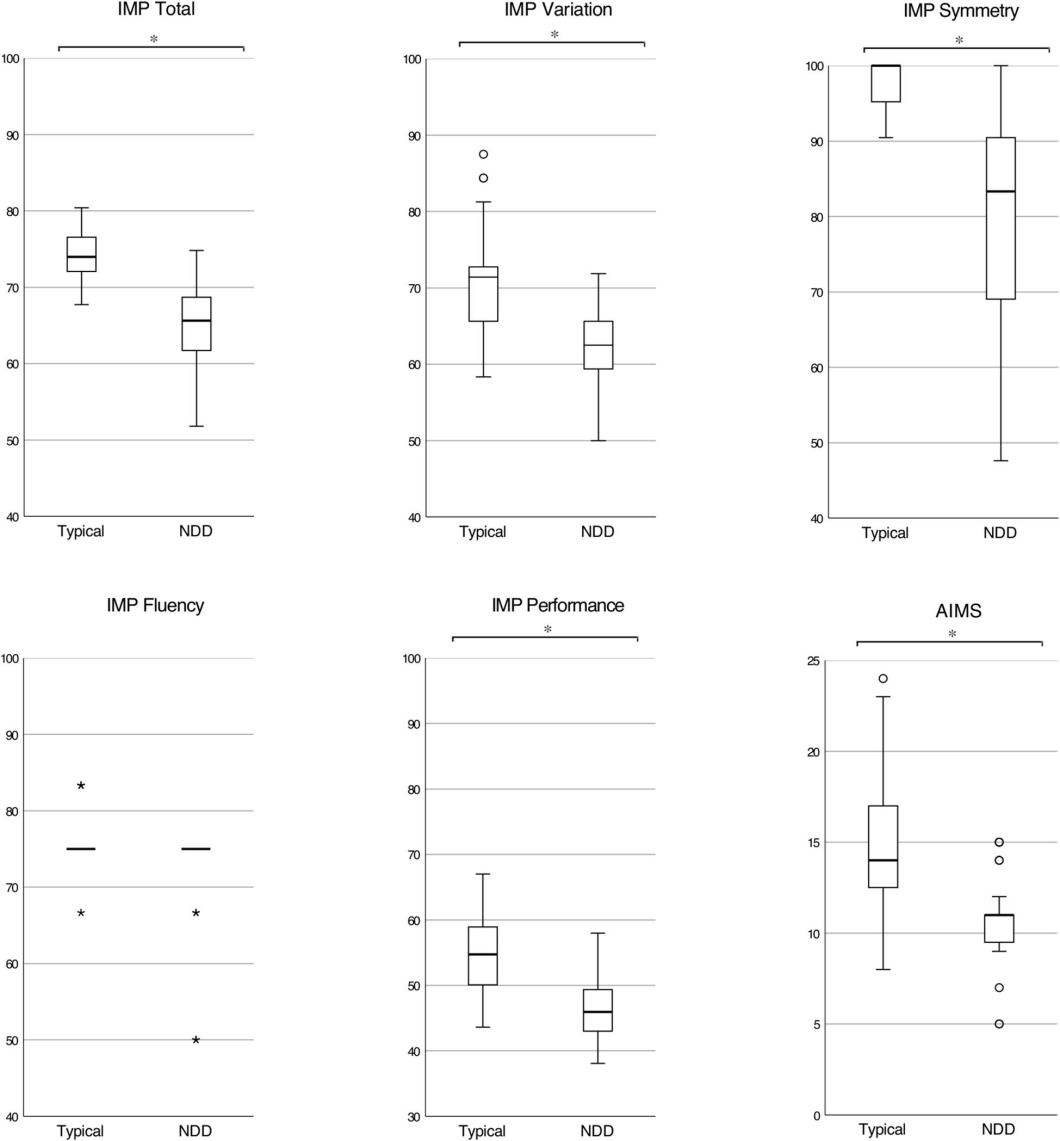
Infant Motor Profile (IMP) scores, Alberta Infant Motor Scale (AIMS) scores at the corrected age of 5 months in children with typical development and neurodevelopmental disorders (NDD). Mann-Whitney U test: *p < .001

**Fig. 3:**
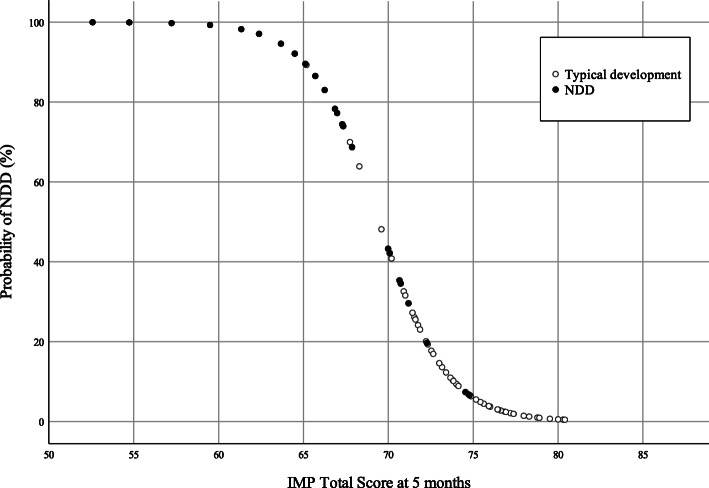
Scatterplot of predicted probability of neurodevelopmental disorders (NDD) from the regression model derived from the Infant Motor Profile (IMP). Total scores at the corrected age of 5 months. Values ≤70 determine a major increase of the probability to develop NDD. Empty markers represent actual typical development, full markers represent actual NDD

The IMP Total score at 5 months showed a highly significant relation with the neurodevelopmental outcome: infants with a typical development showed a substantially higher score (median 74.0; interquartile range 4.6) than infants with NDD (median 65.6; interquartile range 9.1)*; p* < .001 (see Fig. 2)*.* Furthermore, it was confirmed that Variation, Symmetry and Performance were individually correlated with the neurodevelopmental outcome (*p* < .001), as was the AIMS (*p* < .001*)*. In logistic regression, the IMP Total score was confirmed to be the best single predictor of NDD (*p* < .001): the model based on the IMP Total confirmed a good fit (Hosmer-Lemeshow’s *P* = .67) and a good predictive power (Nagelkerke’s R2 = 0.737) with an overall accuracy of classification of 88%. Figure 3 shows a graphical representation of the probability to develop a NDD according to the model based on the IMP Total score values. A similar model based on the AIMS score showed a lower predictive power (Nagelkerke’s R2 = 0.445).

**Fig. 4:**
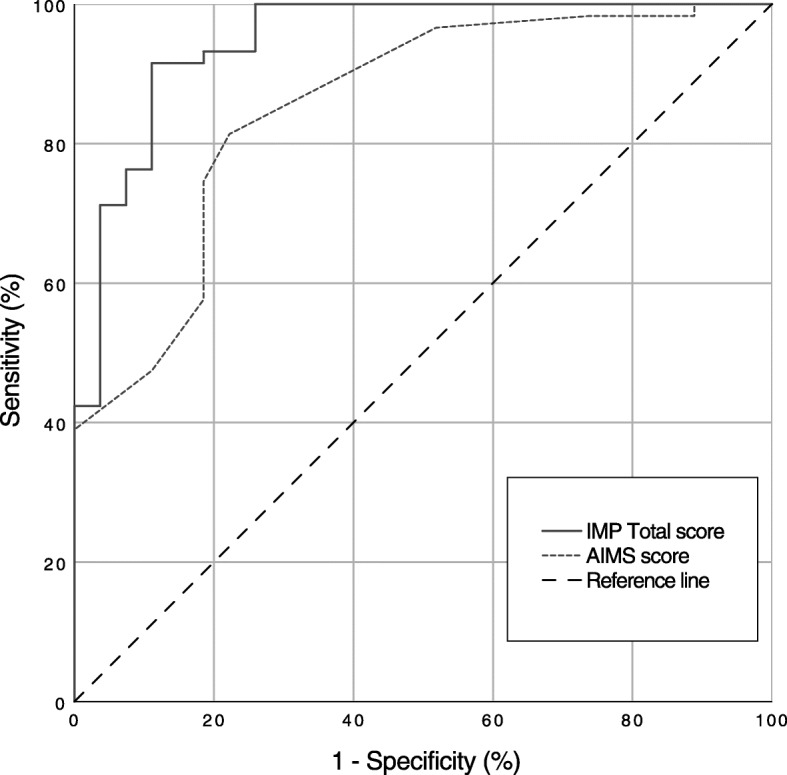
Receiver operating characteristic (ROC) curve of the Infant Motor Profile (IMP) Total score and the Alberta Infant Motor Scale (AIMS) score as predictors of neurodevelopmental disorders (NDD) at the corrected age of 5 months

The ROC curves generated from the IMP Total score and the AIMS Total score are reported in Fig. 4 summarizing the overall diagnostic accuracy of the two assessments. The Area Under the Curve (AUC) for the IMP Total score was outstanding (0.95; *p* < .001; CI95% 0.90–0.99) while the AUC for the AIMS score was lower (0.85; p < .001; CI95% 0.77–0.94) indicating that the accuracy of the IMP is higher in the early detection of NDD. The definition of an optimal cut-off point of 70 allowed us to obtain an overall sensitivity of 93% and a specificity of 81% in the prediction of NDD (PPV 84%; NPV 90%).

Individual ROC curves were developed for each IMP domain: AUC values for the IMP Variation, Symmetry and Performance showed excellent accuracy whereas values for the IMP Fluency indicated poor prediction (see Table 4).

**Table 4:** Area under the ROC curves for IMP Total score and domains score

	Area under the ROC curve (95% CI)	***p*** value
**IMP Total Score**	0.95 (0.90–0.99)	<.001
IMP Variation	0.84 (0.75–0.93)	**<.**001
IMP Fluency	0.59 (0.45–0.72)	.194
IMP Symmetry	0.92 (0.85–0.98)	<.001
IMP Performance	0.81 (0.69–0.92)	<.001
**AIMS Total Score**	0.85 (0.77–0.94)	<.001

## Discussion

Our data confirm the excellent concurrent validity of the IMP and the AIMS. Values are in line with data previously published by Heineman et al. [9] confirming a maximal correlation for the IMP Performance and lower correlation for the IMP Fluency. The highest correlation between the IMP Performance and the AIMS is explainable as both are focused on achievements of motor milestones. The association between the IMP and the GMA was also good, as evidence of the solid construct validity of the IMP. In fact, both assessments reflect the same qualitative elements such as variation, symmetry and fluency of movements.

In the definition of the prognosis of children at risk of NDD, the correlation between clinical and neuroradiological tools is pivotal. In our study, the IMP Total score reflected the presence and the severity of brain injury more accurately then the AIMS. This data supports the idea that any neurological condition which affects the complexity of brain connectivity results in a reduction of the complexity of the motor repertoire [28]. This subtle and complex process is better captured by qualitative assessments such as the IMP rather than performance-based tools such as the AIMS.

We compared the ability of the IMP and the AIMS to predict the neurodevelopmental outcome in a population of infants who had been specifically selected for being at risk of NDD. While both tests were confirmed to be significantly correlated to NDD, the IMP Total score proved to be the most accurate single predictor of an atypical outcome. At 5 months CA, after the identification of a cut-off value of 70, the IMP Total score predicted NDD with high sensitivity (93%) and specificity (81%). Among the different sub-scores, all the domains, except for Fluency, were significantly related to the outcome. IMP Fluency reflects the ability of infants to perform smooth and seamless movements in different conditions (e.g. sitting, supine, walking...). The domain is composed of only 7 items (6 for non-walking infants) which mostly investigate the presence of tremors and non-fluent movements during the assessment. Unlike previously published data [14, 15], the majority of infants in our study sample scored the same low value on this domain (75 points). Moreover, the IMP fluency at 5 months was poorly correlated to the presence of brain injury and showed no significant relation with the neurodevelopmental outcome. A possible reason for this might be related to the different characteristics of our study population which was largely selected among infants who experienced prolonged hospitalizations in NICU. Indeed, if on the one hand lack of fluent movements could be one of the first indicators of non-optimal neurologic development, it is also true that benign shudders, jitteriness and tremors are commonly seen during the first months of life, especially in infants with a prolonged stay in NICU [29, 30]. Furthermore, the small number of items contributing to the IMP Fluency score resulted in a reduced variability of the values.

This is the first study to evaluate the predictive validity of the IMP in a population of at-risk infants, written by a group of researchers who are in no way connected to the developers of the scale. One of its strong points is the presence of three different video-based assessments which were scored by blind assessors, another being the fact that all the infants were recruited at the very early stages of life among infants at risk of NDD. Nevertheless, the study presents several limitations. First of all, the short duration of follow-up and the absence of a structured battery of assessments at 18 months may not have allowed us to identify milder conditions which require more time and standardized assessments for the diagnosis. Infants were retrospectively recruited among the participants of two clinical trials during which different kinds of early intervention programs had been were provided; a mild effect of these programs on the final outcome cannot be ruled out [17, 31]. Furthermore, we provided a coarse classification of brain imaging since no widely used classification system of perinatal brain injury takes into account both term and preterm patterns of injury. Hence, our classification might not accurately reflect the actual severity of some patterns of brain injury. For all these reasons, and for the nature of the retrospective design, the present findings cannot be generalized to all infants at risk of neurodevelopmental disorders. Further research should aim at assessing the predictivity of the IMP in prospective longitudinal studies including more homogeneous populations of infants at risk of NDD.

## Conclusion

The accurate prediction of NDD during the first months of life is paramount in order to provide early access to rehabilitative intervention to children at risk. Literature supports the combined use of the GMA and brain MRI for an early prediction of NDD. However, starting from 4 to 5 months CA general movements gradually disappear, thus leading to the need to find other reliable qualitative assessments of early motor behaviour. The IMP represents a valid alternative; the high flexibility, the absence of need for expensive kit materials and its excellent psychometric performances make the IMP an extremely interesting tool in the evaluation of infants at risk of NDD. In this sense, a greater integration of the IMP among the clinical tools used during the follow-up programs will be useful. Also, the use of the IMP as an outcome measure in clinical trials will provide data on the possible use of this instrument to reflect the effect size of a treatment.

The present study shows that the IMP has a high concurrent correlation with two of the most used clinical assessment tools in early infancy (the AIMS and the GMA). Furthermore, we demonstrated that the IMP accurately reflects the degree of early brain injury and that there is a clear relationship between early motor development assessed with the IMP and neurodevelopmental outcome. These findings support the idea that at the early stages of development, qualitative aspects of motor behaviour may reflect the complexity of cerebral connectivity, thus representing a strong indicator of a future diagnosis of NDD.

Additional observational trials with prospective cohorts of at-risk infants should further elucidate the relationship between early motor behaviour and neurodevelopment, particularly by investigating how different patterns of brain injury affect the different IMP domains.

## Supplementary Information

**Additional file 1: Supplementary Figure 1**. Flow-chart of patients’ enrolment.

## Data Availability

The dataset analysed during the current study is available from the corresponding author to researchers on reasonable request.

## Abbreviations

ADHD: Attention Deficit Hyperactivity Disorder
AUC: Area Under the Curve
GMA: General Movement Assessment
IMP: Infant Motor Profile
MRI: Magnetic Resonance Imaging; CP: Cerebral Palsy
NICU: Neonatal Intensive Care Unit
NDD: Neurodevelopmental Disorder

## References

[1] Thapar A, Cooper M, Rutter M (2017). Neurodevelopmental disorders. Lancet Psychiatry.

[2] Spittle A, Orton J, Anderson P, Boyd R, Doyle LW. Early developmental intervention programmes post-hospital discharge to prevent motor and cognitive impairments in preterm infants. Cochrane Database Syst Rev. 2012;12:1–100. 10.1002/14651858.CD005495.pub3 23235624

[3] Novak I, Morgan C, Adde L, Blackman J, Boyd RN, Brunstrom-Hernandez J (2017). Early, accurate diagnosis and early intervention in cerebral palsy. JAMA Pediatr.

[4] Groen SE, de Blécourt ACE, Postema K, Hadders-Algra M (2005). General movements in early infancy predict neuromotor development at 9 to 12 years of age. Dev Med Child Neurol.

[5] Caesar R, Colditz PB, Cioni G, Boyd RN. Clinical tools used in young infants born very preterm to predict motor and cognitive delay (not cerebral palsy): a systematic review. Dev Med Child Neurol. 2020:1–9. 10.1111/dmcn.14730 33185285

[6] Einspieler C, Bos AF, Libertus ME, Marschik PB. The general movement assessment helps us to identify preterm infants at risk for cognitive dysfunction. Front Psychol. 2016;7:1–8. 10.3389/fpsyg.2016.00406 PMC4801883 27047429

[7] Heineman KR, Hadders-Algra M (2008). Evaluation of neuromotor function in infancy - A systematic review of available methods. J Dev Behav Pediatrics.

[8] Einspieler C, Sigafoos J, Bartl-Pokorny KD, Landa R, Marschik PB, Bölte S (2014). Highlighting the first 5 months of life: general movements in infants later diagnosed with autism spectrum disorder or Rett syndrome. Res Autism Spectr Disord.

[9] Heineman KR, Bos AF, Hadders-Algra M (2008). The infant motor profile: a standardized and qualitative method to assess motor behaviour in infancy. Dev Med Child Neurol.

[10] Edelman GM. Neural Darwinism: selection and reentrant signaling in higher brain function. Neuron. 1993;10(2):115–25. 10.1016/0896-6273(93)90304-a 8094962

[11] Hadders-Algra M. Variation and variability: Key words in human motor development. Phys Ther. 2010;90(12):1823–37. 10.2522/ptj.20100006 20966209

[12] Heineman KR, Schendelaar P, Van den Heuvel ER, Hadders-Algra M (2018). Motor development in infancy is related to cognitive function at 4 years of age. Dev Med Child Neurol.

[13] Heineman KR (2013). Reliability and concurrent validity of the infant motor profile. Dev Med Child Neurol.

[14] Heineman KR, Bos AF, Hadders-Algra M (2011). Infant motor profile and cerebral palsy: promising associations. Dev Med Child Neurol.

[15] Wu YC, Heineman KR, La Bastide-Van Gemert S, Kuiper D, Drenth Olivares M, Hadders-Algra M. Motor behaviour in infancy is associated with neurological, cognitive, and behavioural function of children born to parents with reduced fertility. Dev Med Child Neurol. 2020;62(9):1089–95. 10.1111/dmcn.14520 PMC7496844 32222973

[16] Tveten KM, Hadders-Algra M, Strand LI, Van Iersel PAM, Rieber J, Dragesund T (2020). Intra- and inter-rater reliability of the infant motor profile in infants in primary health care. Phys Occup Ther Pediatr.

[17] Sgandurra G, Beani E, Giampietri M, Rizzi R, Cioni G, Cecchi F (2018). Early intervention at home in infants with congenital brain lesion with CareToy revised: a RCT protocol. BMC Pediatr.

[18] Einspieler C, Prechtl HFR (2005). Prechtl’s assessment of general movements: a diagnostic tool for the functional assessment of the young nervous system. Ment Retard Dev Disabil Res Rev.

[19] Piper MC, Pinnell LE, Darrah J, Maguire T, Byrne PJ (1992). Construction and validatikon of the Alberta infant motor scale (AIMS). Canadian Journal of Public Health.

[20] Spittle AJ, Lee KJ, Spencer-Smith M, Lorefice LE, Anderson PJ, Doyle LW. Accuracy of two motor assessments during the first year of life in preterm infants for predicting motor outcome at preschool age. PLoS One. 2015;10(5):1–15. 10.1371/journal.pone.0125854 PMC4430525 25970619

[21] Darrah J, Piper M, Watt M-J (2008). Assessment of gross motor skills of at-risk infants: predictive validity of the Alberta infant motor scale. Dev Med Child Neurol.

[22] American Psychiatric Association (2013). Diagnostic and Statistical Manual of Mental Disorders, Fifth Edition.

[23] Martinez-Biarge M, Groenendaal F, Kersbergen KJ, Benders MJNL, Foti F, Van Haastert IC (2019). Neurodevelopmental outcomes in preterm infants with white matter injury using a new MRI classification. Neonatology..

[24] Volpe JJ, Inder TE, Darras BT, de Vries LS, du Plessis AJ, Neil JJ (2017). Volpe’s neurology of the newborn. Volpe’s Neurology of the Newborn.

[25] De Vries LS, Groenendaal F (2010). Patterns of neonatal hypoxic-ischaemic brain injury. Neuroradiology..

[26] Portney LG, WM (2020). Foundations of clinical research: applications to evidence-based practice. Foundations of clinical research: Applications to evidence-based practice.

[27] Hosmer DW, Lemeshow S. Applied Logistic Regression Second Edition. Applied Logistic Regression. New York: Wiley; 2004.

[28] Hadders-Algra M (2008). Reduced variability in motor behaviour: an indicator of impaired cerebral connectivity?. Early Hum Dev.

[29] Bonnet C, Roubertie A, Doummar D, Bahi-Buisson N, De Cock VC, Roze E (2010). Developmental and benign movement disorders in childhood. Movement Disorders.

[30] Shuper A, Zalzberg J, Weitz R, Mimouni M (1991). Jitteriness beyond the neonatal period: a benign pattern of movement in infancy. J Child Neurol.

[31] Sgandurra G, Bartalena L, Cioni G, Greisen G, Herskind A, Inguaggiato E (2014). Home-based, early intervention with mechatronic toys for preterm infants at risk of neurodevelopmental disorders (CARETOY): a RCT protocol. BMC Pediatr.

