# Supplementary figures and images for: Concurrent and predictive validity of the infant motor profile in infants at risk of neurodevelopmental disorders
Source: BMC Pediatr. 2021 Feb 6;21:68. doi: 10.1186/s12887-021-02522-5 (PMC7866878; doi:10.1186/s12887-021-02522-5)

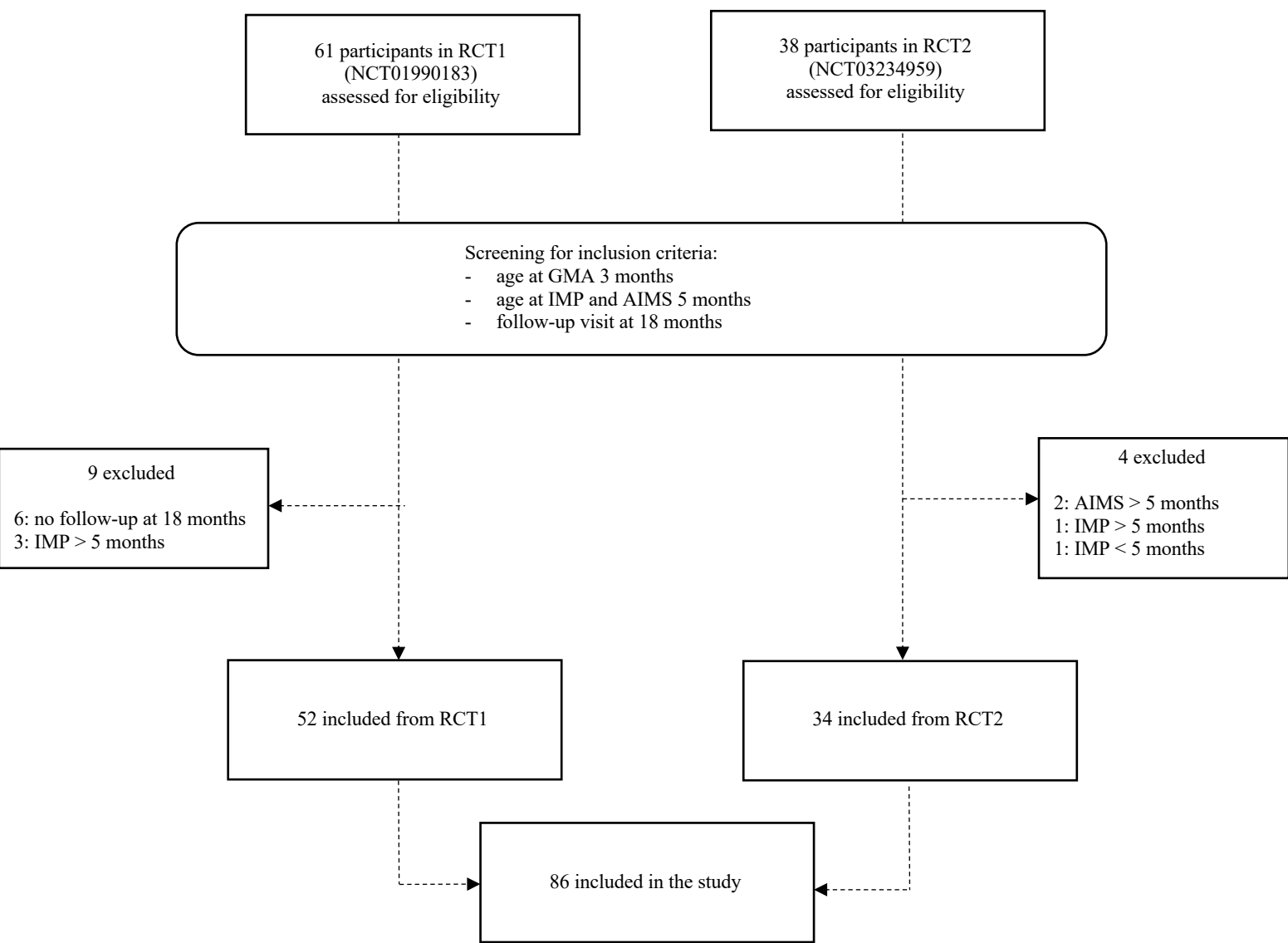

*Supplementary Figure 1: Flow-chart of patients' enrolment.*

Supplement: Supplementary file 1 — Additional file 1: Supplementary Figure 1. Flow-chart of patients’ enrolment. [file 12887_2021_2522_MOESM1_ESM.pdf]
